# eHealth and the Digital Divide Among Older Canadians: Insights from a National Cross-Sectional Study

**DOI:** 10.2196/72274

**Published:** 2025-11-25

**Authors:** Mirou Jaana, Haitham Tamim, Guy Paré

**Affiliations:** 1 Telfer School of Management, University of Ottawa, 55 Laurier Avenue East, Ottawa, ON, K1N 6N5, Canada, 1 6135625800 ext 3400; 2 School of Business, Algonquin College, Ottawa, ON, Canada; 3 Department of Information Technologies, HEC Montréal, Montreal, QC, Canada

**Keywords:** older adults, social determinants, health care disparities, health care system, eHealth, mHealth, digital health, survey

## Abstract

**Background:**

The multidisciplinary life course theory emphasizes the relation between a person’s choices and their socioeconomic context, and their capacity to make decisions within existing opportunities or constraints. Older age is particularly characterized by social and environmental conditions that may impact people’s use of technology and eHealth applications.

**Objective:**

This research aims to present an overview of eHealth application use among older Canadian adults and examine the relationship between eHealth use and social and health system interaction determinants.

**Methods:**

We conducted a national cross-sectional survey of older adults (n=2000) in Canada, assessing their technology (eg, tablets, computers) and eHealth application (eg, fall detection and telemonitoring technologies, internet) use, social determinants (eg, sociodemographic characteristics, environmental living conditions), and health system interactions (eg, health status, access to care, services utilization).

**Results:**

There is technological readiness (owned a computer: 1703/2000, 85.2%; used the internet daily or a few times per week: 1652/2000, 82.6%) among older Canadian adults, although it does not translate into eHealth application use. Internet use to connect with health care professionals, access results or patient portals, or book medical appointments was limited. The use of telemonitoring and fall detection technologies was low (189/2000, 9.4%, and 84/2000, 4.2%, respectively). There were significant variations in eHealth use, highlighting the importance of accounting for social determinants and interactions with the health care system. Of the variance in online access to laboratory results, 12.7% was explained by the province of residence (higher in Ontario and British Columbia), living environment (lower in rural settings), and access or need variables (higher for those with private insurance and willingness to pay for quicker access; higher for those hospitalized). Women reported more internet use for self-diagnosis and looking for online information. Individuals with excellent perceived health and those with no recent emergency visits or home care services reported greater use of mobile health apps and fall detection technologies (odds ratio [OR]=2.16, 95% CI 1.23‐3.80; OR=3.427, 95% CI 1.55‐7.60), respectively. A digital divide exists within the older adult population, which raises concerns about whether those with higher needs and limited resources have access to and can benefit from eHealth applications.

**Conclusions:**

Addressing the digital health gap among older adults is not simply a matter of technological access but also a matter of health equity and system sustainability. Without deliberate policies, digital health risks reinforcing existing disparities by disproportionately excluding those with the greatest health needs and the fewest resources. Our findings identify the groups most at risk of digital exclusion, such as rural residents, institutionalized older adults, and those with limited financial or insurance coverage, and point to where interventions can yield the greatest benefit.

## Introduction

The number of older adults is growing and expected to reach 2.1 billion worldwide by 2050 [1]. In Canada, approximately 18.5% of the population are ≥65 years old, representing more than 7 million Canadians [2], and older Canadians constitute the fastest growing age group [3]. As a result, challenges have emerged with addressing the increasing needs of older adults who utilize health care services more frequently than other age groups [4]. eHealth, which refers to the use of information and communication technologies (ICTs) for health and encompasses a variety of mobile health (mHealth) apps [5,6] and internet use for health services and information delivery [7,8], presents an opportunity to enhance care quality, equity, efficiency, and management of health conditions among older adults [9-11].

In recent years, there has been a growing interest worldwide in studying technology in the context of older adults’ care [12-16]. With the global challenges related to the shortage of providers and the shift of care delivery outside of medical settings, older adults are expected to play a more active role in the management of their health [17]. In addition, as baby boomers move into the “third age” of retirement characterized by searching for new experiences and learning new things, there is an increasing need to assess their knowledge and use of information technologies [18], which can support their health and well-being. This is particularly important in the context of global health care systems estimating an increase in the proportion of older adults [19] and the limited resources available to support them.

The multidisciplinary life course theory (LCT) [20,21] emphasizes the integrated relation between a person’s choices and their socioeconomic context, alongside their capacity to make decisions within existing opportunities and constraints [22]. It is a multidisciplinary framework that integrates factors from various disciplines (eg, sociology, psychology) to understand human behavior. An individual’s family constitutes a “social group” that is embedded in a larger social context [23], and questions related to this factor are at the core of the LCT [21]. The components characterizing the LCT include geographical location, social ties (eg, presence and attributes of family members and societal experiences), stages in life (eg, generational group differences), variability (eg, in gender, social class, education, wealth, family support), and personal control (eg, environmental opportunities and constraints), all of which can present circumstances that shape individuals’ perceptions and decisions [23]. Along the same line, the World Health Organization (WHO) emphasizes the importance of social determinants (ie, environment in which people live, work, age, and can access money and resources), as these can create avoidable differences in health across communities [24]. In addition, the Andersen model for health care utilization emphasizes the importance of enabling factors (eg, access to care) and need factors (eg, health problems) in shaping the utilization of health services [25]. Therefore, interactions with the health system, including factors related to access to resources, services utilization, health status, and perceived needs, represent contextual factors that can influence eHealth use.

Prior research on older adults’ use of technology for web-based socialization discussed the relevance of financial and knowledge barriers, as well as social factors related to family, social motivation, and appropriate environments [12], and the need to investigate social and economic factors that persist as challenges to technology uptake [14]. Aside from a few studies that investigated social determinants in relation to telehealth use by athletic trainers [26], eHealth engagement among people living with HIV [27], eHealth literacy levels in older adults [28], older adults’ perceptions and views on eHealth services [11], and access to and preferences for patient portal use among older adults [29], limited research exists in this area. In Canada, an earlier study with older adults focusing on health tracking behaviors showed that greater than 60% tracked their health manually, thus emphasizing the limited uptake of digital self-tracking in this group compared with the general population [30] and the need to better understand their eHealth use patterns. Hong and Cho [31], who reviewed instruments assessing eHealth behaviors, also called for national surveys adapted to technology development that may be leveraged to analyze eHealth behaviors for informing evidence-based policies.

Despite increasing scholarly interest in older adults’ use of technology, few studies have provided a comprehensive, nationally representative, and theory-driven portrait of eHealth engagement in this population. Most prior work has been limited to localized samples, focused on specific technologies (eg, patient portals, telemonitoring pilots), or relied on instruments not adapted to current technological realities. This lack of evidence impairs the ability of policymakers and health system leaders to design equitable digital health strategies that address the needs of the fastest-growing group of health care users.

Grounded in the LCT and Andersen health care utilization model, this study makes 3 important contributions. First, it provided the first national baseline of eHealth use among older Canadian adults across a broad range of applications, enabling pre- and postpandemic comparisons and international benchmarking. Second, it revealed how social determinants and health care system interactions shape digital health behaviors, offering conceptual insights into the factors that enable or constrain digital engagement in later life. Third, it highlighted equity-relevant gaps, identifying groups at heightened risk of digital exclusion and pointing to where targeted interventions are most urgently needed.

## Methods

### Study Design

A cross-sectional survey of Canadian older adults assessing their use of specific technologies or ICTs, eHealth, and health IT solutions was conducted online using a self-administered computer-assisted web interface (web surveys: 1500/2000, 75%) and by phone using computer-assisted telephone interviews (phone surveys: 500/2000, 25%).

### Settings and Participants

A random national sample of 2000 Canadian residents from all provinces, aged 65 years and older, and who spoke English or French was selected from a proprietary online panel of more than 400,000 households owned by Léger, Canada’s largest and leading research and analytics firm. To ensure a random sample, no quotas were set initially, and the data were weighted after sampling according to gender, age, and region to maximize the representativeness of the Canadian population of older adults.

### Assessments and Data Sources

**Figure 1.**
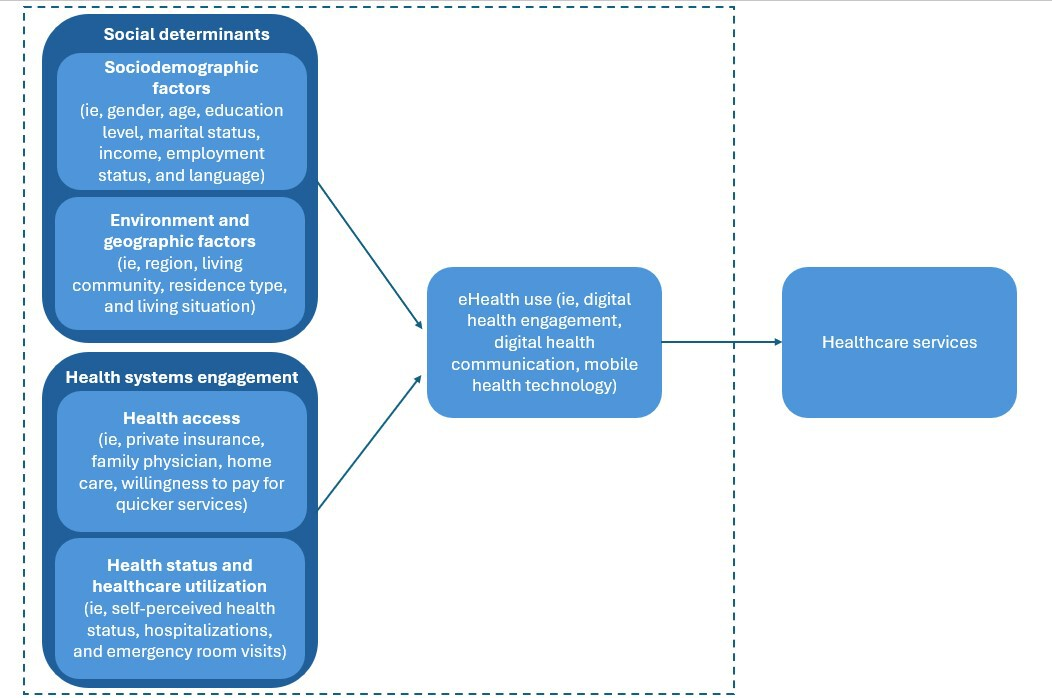
Conceptual framework of social and system-level determinants shaping older adults’ use of eHealth applications.

In line with the conceptual framework shown in Figure 1, the survey instrument included 3 main sections: (1) sociodemographic characteristics, living environment, and health system interaction factors; (2) eHealth and ICT use; and (3) fall detection technology (FDT) and telemonitoring technology (TM) use.

Grounded in the principles of the LCT, sociodemographic variables were measured with standardized indicators including gender, age, marital status, income, education, language, and employment. Questions assessing the living environment included the region (representing provinces), community (rural/suburb/urban/metropolitan), place of residence (home/retirement home/long-term care), and living arrangement (alone/with family/with spouse or partner) to give a comprehensive overview of the older adult social context.

To measure interactions with the health care system, we used the following indicators: private insurance (yes/no), family physician (yes/no), home care (yes/no), willingness to pay for quicker access to services (yes/no), hospitalizations and emergency room visits in the past 6 months (yes/no), and perception of own health (categorical). Satisfaction with the health care system, with access to health care services, and with the health care services received were also included in the survey (categorical).

The outcomes of interest in this study were divided into 3 categories that represent eHealth use: (1) *digital health engagement*, including the extent of internet use for actively searching and accessing health-related information and services online (eg, searching for online information about a health condition, accessing laboratory test results); (2) *digital health communication*, including the extent of willingness or interest in using digital means to communicate about health services (eg, use of email to discuss a health condition with a physician, obtain information about trusted websites); and (3) *mobile health technology use*, including the extent of use of mobile apps, FDTs, and TMs. The questions assessing FDT and TM use were binary (yes/no). The frequencies of use of mobile apps for health and internet for accessing information, resources, and communicating with providers were measured on a 5-point scale (1=Never to 5=Always). Interest or willingness to exchange online information was also assessed on a 5-point scale (1=Not at all to 5=Totally).

The survey instrument was pretested with 47 respondents by phone and on the web. A change was subsequently made to the skip pattern for one of the questions. Data collection was completed over a 3-week period in 2018. Although the data are not very recent, they provide a robust baseline for pre- and post–COVID-19 pandemic comparisons, especially in the absence of data on eHealth use among older adults.

### Data Analysis

Descriptive data analysis was conducted to gain an understanding of the profile of older adults and their technology-related behaviors. Bivariate nonparametric tests were used for analyses of associations between continuous dependent variables (eg, search online for information) and categorical independent variables (eg, age, education). Specifically, nonparametric 2-independent samples analyses (Mann-Whitney) were conducted for independent variables with only 2 categories, and nonparametric K-independent samples analyses (Kruskal-Wallis) were conducted for independent variables with more than 2 categories. *χ*^2^ and Fischer’s exact tests for categorical variables were used to examine the associations between the binary dependent variables related to mobile technology use and the determinants (eg, sociodemographic characteristics, living environment) and interactions with the health system variables. Multivariate regression analysis for eHealth scale questions and binary logistic regression analysis for binary mHealth technology questions (TM and FDT) were performed to examine the significant relationships between eHealth use and the social and health system interaction variables that showed significant associations at the bivariate analysis level. To assess potential multicollinearity among the independent variables for the multivariate analysis, we calculated the variance inflation factor (VIF) for each predictor. All independent variables, with the exception of marital status (ie, “married”; VIF~10), had a VIF lower than the general threshold of 10 [32]. Therefore, we retained all theoretically relevant variables to maintain the breadth of the models, which was the main aim with our modeling approach.

### Ethical Considerations

Ethics approval was granted by the University of Ottawa Ethics Research Board, and all data were anonymized. Eligible respondents were provided with an information letter that explained the scope and purpose of the project. It specified that their participation was voluntary and completing the questionnaire indicated their consent for participating in this study. Léger panelists are rewarded for their participation over time using a series of financial incentives that can be accumulated and cashed out or donated for a charitable cause. Participants in this survey who completed this survey were rewarded 1200 Léger Opinion points (CAD $20=20,000 points; a currency exchange rate of CAD $1=US $0.71 is applicable) that can be redeemed through their preferred reward method.

## Results

### Sample Characteristics

Table 1 reveals the variations in social determinants and living conditions. The majority of respondents were 65 years to 69 years old (663/2000, 33.2%), were women (1092/2000, 54.6%), were married (1201/2000, 60.1%), had a college or university degree (1243/2000, 62.2%), spoke English at home (1496/2000, 74.8%), earned less than CAD $75,000 (1184/2000, 59.2%), and lived in Ontario (760/2000, 38%) or Quebec (505/2000, 25%), which are the most populous and largest health care jurisdictions in the country. In addition, 68.5% (1369/2000) lived in metropolitan cities or suburbs, and 96.1% (1922/2000) lived in their own homes or apartments; one-third (665/2000, 33.3%) lived alone.

**Table 1.** Study sample sociodemographic characteristics and interaction patterns with the health care system (n=2000).

Characteristics and interaction patterns	Results
Gender, n (%)	
Male	908 (45.4)
Female	1092 (54.6)
Age (years), n (%)	
65-69	663 (33.2)
70-74	479 (23.9)
75-79	360 (18)
80-84	323 (16.2)
≥85	175 (8.7)
Highest education level, n (%)	
Elementary school	42 (2.1)
Intermediate school	56 (2.8)
High school	630 (31.5)
College degree	523 (26.2)
University, undergraduate	476 (23.8)
University, graduate degree	244 (12.2)
Other	28 (1.4)
Marital status, n (%)	
Single	141 (7)
Married	1201 (60.1)
Widowed	401 (20.1)
Separated/divorced	248 (12.4)
Other	9 (0.4)
Income (CAD $^a^), n (%)	
<25,000	202 (10.1)
25,000-49,999	589 (29.4)
50,000-74,999	393 (19.6)
75,000-99,999	270 (13.5)
100,000-124,999	122 (6.1)
≥125,000	61 (3.1)
I prefer not to answer	363 (18.2)
Employment, n (%)	
Employed, full-time	77 (3.8)
Employed, part-time	94 (4.7)
Retired	1790 (89.5)
Other	39 (1.9)
Home language, n (%)	
French	459 (23)
English	1496 (74.8)
Other	45 (2.2)
Region, n (%)	
Prairies (Alberta, Saskatchewan, Manitoba)	293 (14.7)
British Columbia	287 (14.3)
Maritimes (New Brunswick, Nova Scotia, Prince Edward Island)	126 (6.3)
Newfoundland and Labrador	30 (1.5)
Ontario	760 (38)
Quebec	505 (25.2)
Residential community, n (%)	
Rural (<2500 persons)	306 (15.3)
Small town (2500‐10,000 persons)	306 (15.3)
Suburb (10,000‐50,000 persons)	479 (23.9)
Metropolitan (>50,000 persons)	890 (44.5)
I don’t know	19 (1)
Residence type, n (%)	
My home or apartment	1922 (96.1)
A retirement home	76 (3.8)
Other	2 (0.1)
Living situation, n (%)	
Alone	665 (33.3)
With your wife/husband/partner	1205 (60.3)
With family or friends	119 (5.9)
Other	11 (0.5)
Private insurance, n (%)	
Yes	1098 (54.9)
No	902 (45.1)
Family physician, n (%)	
Yes	1896 (94.8)
No	104 (5.2)
Home care, n (%)	
Yes	83 (4.1)
No	1917 (95.9)
Willingness to pay for quicker access, n (%)	
Yes	647 (32.4)
No	1353 (67.6)
Perception of own health, n (%)	
Excellent	242 (12.1)
Very good	694 (34.7)
Good	725 (36.2)
Fair	281 (14)
Poor	59 (2.9)
Hospitalization (past 6 months), n (%)	
Yes	164 (8.2)
No	1836 (91.8)
Number of hospitalizations (past 6 months), median (range; IQR)^b^	1 (1-6; 1-1)
Emergency room visits (past 6 months), n (%)	
Yes	278 (13.9)
No	1717 (85.9)
I don’t know	5 (0.2)
Number of emergency visits (past 6 months)^c^, median (range; IQR)	1 (1-8; 1-2)

a A currency exchange rate of CAD $1=US $0.71 is applicable.

b Of 164 older adults who reported being hospitalized.

c Of 278 older adults who reported emergency room visits.

### Health Care System Interaction

Of the repondents, who normally have access to a national health insurance, 54.9% (1098/2000) reported having private insurance (Table 1). The majority had a regular family physician (1896/2000, 94.8%), did not receive home care services (1917/2000, 95.9%), and reported good to excellent health (1661/2000, 83.1%). In addition, 8.2% (164/2000) and 13.9% (278/2000) had hospitalizations and emergency visits, respectively, in the past 6 months, and one-third (647/2000, 32.4%) were willing to pay out of pocket for quicker access to services. Overall, 61.8% (1237/2000) reported being diagnosed with one or more chronic conditions, and 13.7% (275/2000) indicated having fallen in the past 6 months (falls: median 1, IQR).

Overall, the satisfaction of respondents with the *health care system* was high (very satisfied: 366/2000, 18.3%; satisfied: 1086/2000, 54.3%). In addition, 79.1% (1583/2000) indicated good to high satisfaction with *access to health care services*, and 84.4% (1689/2000) were satisfied to very satisfied with the *health care services received over the past 2 years*.

### ICT and eHealth

The vast majority of surveyed older adults owned a computer (1703/2000, 85.2%), 57.7% (1153/2000) reported having a tablet or iPad, and 53.9% (1077/2000) reported having a smartphone (Multimedia Appendix 1). Fewer owned wearables or mobile devices (238/2000, 11.9%). In addition, 90.3% (1654/2000) reported using email, 50.1% (917/2000) used phone text messaging (eg, WhatsApp, Messenger), and 53.6% (983/2000) used Facebook. Except for a small percent of respondents (238/2000, 11.9%), participants in our study confirmed having used the internet over the past 6 months, mostly daily (1472/2000, 83.6%) or a few times a week (180/2000, 10.2%).

Despite frequent internet and email use and high prevalence of ICTs, the use of internet to connect with a health care professional, access test results or patient portal, or book a medical appointment was limited, although moderate use was reported for using the internet to search for online information about health conditions (median 3, IQR 2-3 on the 5-point Likert scale; Multimedia Appendix 1). The respondents expressed low willingness to use email to exchange information about their health with their health care professionals (median 2, IQR 1-3 on the 5-point Likert scale) and a moderate interest in obtaining information about trusted websites relevant to their health condition and accessing their medical records (median 3, IQR 1-5 on the 5-point Likert scale).

The prevalence of use of TMs and FDTs was low (189/2000, 9.4% and 84/2000, 4.2%, respectively), despite familiarity with these technologies. Among the respondents who had previously or were currently using TMs and FDTs, 81% (153/189) and 86% (72/84), respectively, indicated their willingness to use these technologies again in the future, which indicates satisfaction with these technologies.

### Social Determinants, Health Care System Interaction, and eHealth Use

Bivariate analyses showed significant associations (*P*<.05) between sociodemographic and living environment characteristics and the majority of questions assessing eHealth use (Table 2). For example, being a man, in the younger age group (ie, 65‐69 years), and holding a graduate university degree were significantly associated with a higher willingness or interest in using the internet to access medical records, exchange medical information with family physicians or health care providers, and obtain information on trusted websites to consult about one’s condition (all *P*<.001). On the other hand, being a woman, being separated or divorced, and having a lower income were significantly associated with higher frequency of internet use to search for online information about health problems (all *P*<.001) and the tendency to self-diagnose (*P*<.001, *P*=.02, and *P*=.03, respectively). Living in metropolitan areas and in one’s own home were significantly associated with higher frequency of using mobile apps for health (*P*=.03) and TM use (*P*=.04). Being married or having a partner was also significantly associated with using mobile apps for health (*P*=.01) and FDT use (*P*=.01).

**Table 2.** Bivariate associations between social determinants and eHealth use among older adults in the study sample (n=2000), with *P*<.05 considered statistically significant.

eHealth use	Sex	Age	Education	Marital status	Employment	Income	Region	Language	Community	Live in...	Live with...
Internet use to..., on a scale from 1 to 5 (1=Never; 5=Always), *P* value											
Search online for information	<.001	.13	<.001	<.001	.66	<.001	.03	.14	.001	.46	.19
Self-diagnose	<.001	.001	.03	.02	.049	.03	.07	.001	.009	.83	.49
Access lab results	.40	.20	.11	<.001	.045	<.001	<.001	.003	<.001	.20	<.001
Access patient portal/EMR^a^	.24	.051	.68	.009	.20	.34	<.001	.16	.03	.83	.005
Book appointment	.002	.15	<.001	.02	.69	.004	.001	.20	<.001	.20	.004
Participate in discussion forums	.44	.01	.66	.84	.20	.39	.36	.005	.67	.31	.70
Willingness/interest in..., on a scale from 1 to 5 (1=Not at all; 5=Totally), *P* value											
Use email to discuss health	<.001	<.001	<.001	<.001	.04	<.001	<.001	.04	.19	<.001	<.001
Obtaining information on trusted websites	<.001	<.001	<.001	<.001	<.001	<.001	<.001	.39	<.001	<.001	<.001
Accessing online medical records	<.001	<.001	<.001	<.001	.04	<.001	<.001	.033	<.001	<.001	<.001
Use of…, on a scale from 1 to 5 (1=Not at all; 5=Totally), *P* value											
Mobile apps	≥.99	.007	.11	.01	.74	.002	.007	.16	<.001	.21	.03
Use of… (yes/no), *P* value											
Wearables	≥.99	.95	.87	.35	.89	≥.99	.73	.66	.64	.82	.10
TM^b^	.25	.19	.46	.76	.91	.59	.63	.03	.03	.04	.63
FDT^c^	.11	.01	.55	.01	.95	.52	.99	.54	.67	.004	.03

a EMR: electronic medical record.

b TM: telemonitoring technologies.

c FDT: fall detection technologies.

When examining the relationship between interactions with the health care system and eHealth use (Table 3), having private insurance and a willingness to pay out of pocket for quicker access to health care services were significantly associated with most outcome variables (eg, most *P*≤.001). Respondents who indicated not receiving home care services and no hospitalizations nor emergency visits reported more FDT use (*P*<.001, *P*=.002, and *P*=.002, respectively). Those having an excellent perception of health reported more use of mobile apps for health, higher frequency of internet use to participate in discussion forums about their health, and more willingness or interest in using email to exchange medical information with their physician about their condition.

**Table 3.** Bivariate associations between health care system–related variables and eHealth use among older adults in the study sample (n=2000), with *P*<.05 considered statistically significant.

eHealth use	Family physician	Private insurance	Home care services	Pay for quicker access	Perception of health	Hospitalizations	Emergency visits
Internet use to..., on a scale from 1 to 5 (1=Never; 5=Always), *P* value							
Search online for information	.17	.001	.01	<.001	.43	.56	.004
Self-diagnose	.09	.20	.02	<.001	.29	.60	.80
Ask health care professional	.18	.04	.19	<.001	.35	.67	.58
Access lab results	.04	.008	.77	<.001	.005	.002	.20
Access patient portal/EMR^a^	.02	.02	.54	<.001	.27	.002	.04
Book appointment	.005	.004	.63	.001	.92	.12	.07
Participate in discussion forums	.90	.25	.66	.02	.03	.78	.76
Willingness/interest in..., on a scale from 1 to 5 (1=Not at all; 5=Totally), *P* value							
Use email to discuss health	.67	.001	.002	<.001	.04	.16	.87
Obtaining information on a trusted website	.26	<.001	.001	<.001	.94	.046	.46
Accessing online medical records	.09	<.001	.002	<.001	.48	.12	.98
Use of…, on a scale from 1 to 5 (1=Never; 5=Always), *P* value							
Mobile apps for health	.03	<.001	.85	<.001	.03	.40	.44
Use of… (yes/no), *P* value							
Wearables	.54	.48	.09	.81	.52	≥.99	≥.99
TM^b^	≥.99	.06	.13	.27	.33	≥.99	.78
FDT^c^	.58	.82	<.001	.81	07	.002	.002

a EMR: electronic medical record.

b TM: telemonitoring technologies.

c FDT: fall detection technologies.

### Multivariate Analysis

Multivariate analyses examined the relationship between eHealth use and the sociodemographic characteristics and interactions with the health care system variables that were significant at the bivariate analysis level while controlling for other variables. Table 4 presents which relationships were significant (*P*<.05) in the multivariate analyses; the detailed results (standardized coefficients, odds ratios [ORs], confidence intervals, and *P* values) are presented in Multimedia Appendix 2.

**Table 4.** Multivariate analysis of significant bivariate associations between social determinants and eHealth use among older adults in the study sample.

eHealth use	Sex	Age	Education	Marital status	Employment	Income	Region	Language	Community	Live in...	Live with...
Internet use to..., on a scale from 1 to 5 (1=Never; 5=Always)^a^											
Search online for information	✓^b^		✓			✓	✓		✓		
Self-diagnose	✓	✓		✓				✓	✓		
Ask health care professional		✓	✓				✓				
Access lab results					✓		✓		✓		
Access patient portal/EMR^c^							✓		✓		
Book appointment			✓				✓		✓		
Participate in discussion forums								✓			
Willingness/interest in..., on a scale from 1 to 5 (1=Not at all; 5=Totally)^a^											
Use email to discuss health		✓	✓			✓	✓	✓			
Obtaining information on trusted websites		✓	✓	✓		✓	✓		✓		
Accessing online medical records		✓	✓	✓		✓	✓		✓	✓	
Use of…, on a scale from 1 to 5 (1=Never; 5=Always)^a^											
Mobile apps for health		✓					✓		✓		
Use of… (yes/no)^d^											
TM^e^											
FDT^f^										*✓*	

a Linear regression analyses.

b Significant association (*P*<.05).

c EMR: electronic medical record.

d Logistic regression analyses.

e TM: telemonitoring technologies.

f FDT: fall detection technologies.

eHealth use (ie, digital health engagement and digital health communication) was significantly associated with several sociodemographic variables (Multimedia Appendix 2). Women reported more internet use to search for online information about health problems (*β*=0.148, *P*<.001) and self-diagnosis (*β*=0.079, *P*=.001), whereas older age (≥85 years) was consistently associated with lower frequency of eHealth use (internet use for self-diagnosing, interest in obtaining information about trusted websites for their health condition, accessing online medical records, and use of mobile apps for health). Interestingly, respondents with a lower income indicated a higher frequency of searching for online information about health conditions or problems (*β*=–0.122 for those earning between CAD $50,000 and CAD $75,000 compared with those earning less than CAD $25,000; *P*=.007), and English-speaking respondents (compared with their French-speaking counterparts) had a higher frequency of self-diagnosing (*β*=0.098, *P*<.001) and participating in online forums to discuss aspects related to their health (*β*=0.051, *P*=.04).

Variation in eHealth use was significantly associated with the region and community of residence. Compared with rural areas, living in the suburbs or metropolitan areas was consistently associated with a higher frequency of using mobile apps for health (*β*=0.135, *P*<.001 for metropolitan areas) and internet use for looking for information about health conditions (*β*=0.130, *P*<.001 for suburban areas), self-diagnosing (*β*=0.114, *P*<.001 for suburban areas), accessing laboratory results (suburban areas: *β*=0.092, *P*=.006; metropolitan areas: *β*=0.077, *P*=.03), accessing patient portals (suburban areas: *β*=0.088, *P*=.01; metropolitan areas: *β*=0.077, *P*=.35), and booking appointments online (*β*=0.105, *P*=.005 for metropolitan areas). Residing in retirement homes as opposed to one’s own home was significantly associated with more FDT use (OR=0.366, 95% CI 0.145‐0.923).

There were considerable differences across provinces in Canada, which may be attributed to systemic variation in availability of digital health services leading to variable access and eHealth use. When compared with persons from central Canada (ie, Prairies), Canadians residing in British Columbia reported a higher frequency of using the internet to ask health care professionals about their health (*β*=0.062, *P*=.04) and mobile apps for health (*β*=0.064, *P*=.04) and more willingness or interest in using email to exchange information about their health condition with a physician (*β*=0.087, *P*=.002) and access their online medical records (*β*=0.083, *P*=.002). Residents of Ontario also reported a higher frequency of using the internet to search for health information about their conditions (*β*=0.075, *P*=.03) and access laboratory results (*β*=0.297, *P*<.001) and patient portals (*β*=0.132, *P*<.001), whereas respondents from the Maritimes provinces were more interested or willing to use the internet to email their physicians about their health condition (*β*=0.093, *P*<.001), obtain information on trusted websites to consult about their conditions (*β*=0.062, *P*=.01), and access their online medical records (*β*=0.089, *P*<.001). Current use of the internet to access patient portals (*β*=0.082, *P*=.01) and interest in accessing online medical records (*β*=0.120, *P*=.005) were also higher for older adults in Quebec compared with residents of central Canada.

The enabling and need factors related to the interactions with the health care system investigated in this study revealed a pattern of association with eHealth use. Among the enabling factors, willingness to pay out of pocket for quicker access to health care services and having private insurance were consistently and significantly related to a higher frequency of eHealth use. Specifically, willingness to pay out of pocket was significantly associated with higher frequency of eHealth use for all the measures, with the exception of mobile app and FDT use (Table 5). Respondents who did not have private insurance reported lower frequency for searching for information about their health problem or condition (*β*=–0.054, *P*=.03) and online access to laboratory results (*β*=–0.055, *P*=.02) and patient portals (*β*=–0.063, *P*=.009). With regard to the need variables, older adults who did not have emergency visits in the past 6 months (ie, less needs) reported a significantly lower frequency of searching for information about their health problem or condition (*β*=–0.086, *P*<.001) and accessing patient portals (*β*=–0.058, *P*=.02) but more FDT use (OR=2.16, 95% CI 1.228‐3.800). However, those who did not receive home care services indicated a higher frequency of searching for information about their health problem or condition (*β*=0.052, *P*=.03) and more FDT use (OR=3.427, 95% CI 1.550‐7.596) compared with those receiving home services. Last, a high perceived health status (as excellent) was significantly associated with more frequent mobile app use as opposed to a fair (*β*=–0.073, *P*=.02) or good (*β*=–0.074, *P*=.049) perception of health.

**Table 5.** Multivariate analysis of significant bivariate associations between health care system interaction variables and eHealth use among older adults in the study sample.

eHealth use	Family physician	Private insurance	Home care services	Pay for quicker access	Perception of health	Emergency visits	Hospitalizations
Internet use to..., on a scale from 1 to 5 (1=Never; 5=Always)^a^							
Search online for information		✓^b^	✓	✓		✓	
Self-diagnose				✓			
Ask health care professional				✓			
Access lab results		✓		✓			✓
Access patient portal/EMR^c^		✓		✓		✓	✓
Book appointment	✓			✓			
Participate in discussion forums				✓	✓		
Willingness/interest in..., on a scale from 1 to 5 (1=Not at all; 5=Totally)^a^							
Use email to discuss health				✓			
Obtaining information on trusted websites				✓			
Accessing online medical records				✓			
Use of…, on a scale from 1 to 5 (1=Never; 5=Always)^a^							
Mobile apps for health					✓		
Use of… (yes/no)^d^							
TM^e^							
FDT^f^			✓			✓	

a Linear regression analyses.

b Significant association (*P*<.05).

c EMR: electronic medical record.

d Logistic regression analyses.

e TM: telemonitoring technologies.

f FDT: fall detection technologies.

## Discussion

### Principal Findings

This national survey provides the first comprehensive portrait of eHealth use among older Canadians, offering a valuable baseline for ongoing monitoring and international comparisons. Although the analyses incorporated a broad range of social and health system determinants, our goal was to capture the multidimensional nature of digital engagement and application use in later life, which sets the stage for future specific and targeted investigations. To enhance clarity, the *Discussion* section highlights the most important findings and their implications.

First, although the vast majority of older adults owned digital devices and reported frequent internet use, their actual engagement with eHealth tools remained limited. Use of TMs, FDTs, patient portals, and online appointment systems was low across the sample. This disconnect suggests that access alone is insufficient; awareness, perceived usefulness, and provider support are critical enablers. Importantly, those who had used TMs and FDTs expressed high willingness to use them again, underscoring the importance of initial exposure and positive experiences.

Second, consistent with prior literature, eHealth use was stratified by sociodemographic advantage. Younger age, higher income, and residence in metropolitan areas were associated with greater engagement, while older age, lower income, rural residence, and institutional living environments were linked to reduced use. These patterns reveal persistent inequities within the older adult population, even in a context of high technological readiness. Targeted efforts are needed to ensure that those with the greatest health needs and fewest resources are not left behind.

Third, interactions with the health care system emerged as powerful predictors of eHealth use. Having private insurance, willingness to pay for quicker access, and better perceived health were all associated with higher digital engagement. Conversely, those who had received home care services or experienced recent emergency visits were less likely to use digital tools. This pattern indicates that eHealth may currently serve those with fewer immediate health needs and greater resources, rather than those most in need of coordinated care. Integrating digital health into routine pathways, rather than leaving it as an “optional extra,” is essential for equity.

Fourth, women reported greater use of the internet for health information and self-diagnosis. Although our data do not allow us to assess outcomes such as health anxiety, prior research has suggested that extensive online searching can sometimes heighten distress (ie, “cyberchondria”) [33,34]. This represents a possible area for future investigation.

Several additional associations were observed, including the roles of education, language, and provincial differences, that further illuminate the diversity of older adults’ digital health engagement, which was quite variable across the country. For example, residents of Ontario and British Columbia reported higher use of patient portals and access to laboratory results, while those in the Maritimes expressed greater willingness to communicate with providers online. These contextual nuances reinforce the importance of tailoring digital health strategies to local and cultural environments.

### Comparison With Studies in Other Jurisdictions

Our findings contribute to a growing body of international literature on digital health use among older adults and offer several contrasts with studies conducted in the United States and Europe.

First, older Canadian adults in our sample reported higher levels of interest and perceived usefulness of digital health tools across age groups, including those older than 75 years. This contrasts with US and European studies that consistently report lower adoption rates among the oldest and most socioeconomically disadvantaged groups [35-37]. Second, although previous studies often focus on access and skill gaps, the so-called first- and second-level digital divides, our findings highlight a third dimension: motivational readiness. Many respondents expressed a willingness to use digital tools despite limited experience, particularly when they felt supported by the health care system. This dimension was less frequently emphasized in prior large-scale survey studies [38]. Third, the Canadian context appears to moderate some of the demographic divides found elsewhere. For example, racial and ethnic disparities in digital health use reported in the United States [37] were not observed in our sample, potentially reflecting Canada’s publicly funded and more equity-oriented health care system. Finally, our data indicate relatively high willingness to reuse certain digital health technologies, particularly TMs and various mobile apps. This is an encouraging finding given the infrastructural and digital literacy barriers reported in many European contexts [39,36] and suggests that, when appropriately introduced and supported, older adults can develop positive experiences with digital health tools, even in rural areas.

Taken together, our findings suggest that older Canadian adults are not uniformly resistant to digital health but rather face a complex set of motivational, attitudinal, and structural barriers. Understanding these factors in light of international comparisons can inform more targeted, inclusive, and equity-driven eHealth policies.

### Study Implications and Avenues for Future Research

The findings of this national survey of older Canadians have several implications for policy, practice, and future research on digital health equity. First, the results underscore the need to move beyond binary notions of access (eg, having internet or not) to understand the *multidimensional nature of digital engagement*. Although structural factors such as age, education, and income remain important, our findings reveal that attitudinal variables such as perceived usefulness, trust in digital tools, and self-efficacy are equally, if not more, influential in predicting eHealth adoption. This suggests that interventions should not only address material barriers but also focus on building digital confidence and relevance in health contexts.

Second, the relatively high levels of expressed interest in eHealth technologies among Canadian older adults, even those with limited prior experience, challenge persistent narratives of older adults as digitally disengaged or resistant. This observation contrasts with trends reported in European contexts [35,36], where digital disengagement remains widespread, particularly among the oldest-old, women, and rural residents. Similarly, in the United States, studies by James et al [37] and Schuster et al [40] identified persistent digital divides by race and mental health status. In contrast, our findings point to a more complex and optimistic outlook in Canada, where universal health care, relatively equitable access to health services, and targeted digital health initiatives may be shaping more inclusive digital health trajectories.

Third, the Canadian context provides a unique lens to understand how publicly funded health care systems may buffer against some of the exclusionary forces observed in more market-based systems. For instance, unlike in the United States, where the use of digital health tools is often mediated by private insurance coverage or provider-specific platforms, Canadian respondents interact with a publicly funded system that, in principle, offers more universal access to tools such as e-prescriptions and teleconsultation portals. However, our findings indicate that actual use of digital services like these remains limited, suggesting that accessibility alone does not ensure engagement and that awareness, support, and perceived usefulness remain critical enablers.

Based on these findings, several avenues for future research emerge. Longitudinal studies are needed to assess how digital engagement evolves over time among older adults, especially as younger cohorts with greater baseline digital skills age into retirement. Moreover, future research should examine how *relational dimensions*, such as the support of health care professionals, caregivers, and peers, mediate eHealth use among older adults. Comparative studies across health systems and sociopolitical contexts would further elucidate the interplay of systemic and individual-level factors in shaping digital engagement. Building on these findings, targeted investigation of specific eHealth applications using mixed methods and qualitative approaches would provide a more in-depth understanding of the interaction of factors influencing their use. Finally, there is a need to develop and evaluate *intervention strategies* that go beyond digital literacy training. These should include motivational and psychosocial components, co-designed with older adults, to enhance perceived value and usability of digital tools in real-life care scenarios. Integrating such approaches into existing health care pathways can ensure that digital transformation in health systems is truly inclusive, responsive, and sustainable for an aging society.

### Study Limitations

It is important to note some limitations associated with this research. The cross-sectional nature of this study precludes a thorough assessment of causal relationships between the social determinants and variables related to the interactions with the health care system in relation to eHealth use. For example, the odds of using FDTs were considerably higher among older adults who did not receive home care services nor were hospitalized in the past 6 months. However, it was not possible to determine whether FDT use precluded the need for home care and prevented hospitalizations or whether FDT was used due to the absence of home services and better health.

The respondents’ profile points to a relatively high level of education (62% had a college degree or higher) with the majority residing in their own homes and not alone, living in suburbs or metropolitan areas, having a regular family physician, and having private health insurance (ie, generally good access to a broad range of health care services despite limited use of home care services). Thus, we may expect a lower level of eHealth penetration among the broader older adult population, which further underscores the current suboptimal benefits that older adults are gaining from these technologies.

Since the data were collected from a single country, the generalizability of the results is limited unless the survey is replicated in other contexts. The online nature of the survey, although complemented with phone surveys, may still have excluded potential respondents who did not have access to the internet or phone calls. In addition, the closed-ended questions included in the survey did not allow us to fully uncover the reasons behind some of the association patterns that were observed.

When assessing multicollinearity, marital status (ie, “married”) had a VIF close to 10, indicating potential multicollinearity with other predictors in the models. This is expected, as marital status is closely associated with other sociodemographic variables like cohabitation. Although this multicollinearity may affect the precision of the estimated effect of marital status (ie, compresses the *ß* coefficient and makes it more likely to find false negatives or more conservative results), it does not bias the model overall.

Last, we must stress that there are persistent challenges with collecting comprehensive data from older adult populations [38,41,42]. Although the data from this study are not very recent, they provide a baseline for future studies assessing changes in eHealth use among older adults post-COVID-19 pandemic. In addition, since the eHealth construct’s evolution is gradual and eHealth use among Canadian citizens in general is reported to be changing slowly [43], the findings continue to be relevant and present a foundation for longitudinal comparisons.

### Conclusion

This study offers the first comprehensive national assessment of eHealth use among older Canadian adults and provides a valuable baseline for ongoing monitoring and international benchmarking, including pre- and postpandemic comparisons. Our findings highlight the importance of accounting for social determinants and interactions with the health care system when investigating eHealth use in this population.

Our findings are also relevant beyond Canada. They demonstrate how universal health care systems mitigate, but do not eliminate, digital divides, offering lessons for other jurisdictions seeking to advance inclusive digital health strategies. The study reveals a new dimension of the digital divide, namely motivational readiness, which complements traditional access- and skill-based divides. Recognizing this attitudinal and relational component shifts the focus of interventions from infrastructure alone to trust-building, perceived usefulness, and provider support, thereby broadening the policy tool kit for promoting digital equity in aging societies.

## Supplementary material

10.2196/72274 Multimedia Appendix 1 Information and communication technologies (ICT) and patterns of eHealth use among older adults in the study sample.

10.2196/72274 Multimedia Appendix 2 Multivariate analysis results of factors associated with eHealth use among older adults in the study sample.

## Abbreviations

FDT: fall detection technology
ICT: information and communication technology
LCT: life course theory
mHealth: mobile health
OR: odds ratio
TM: telemonitoring technology
VIF: variance inflation factor
WHO: World Health Organization
