# Supplementary material for: eHealth and the Digital Divide Among Older Canadians: Insights from a National Cross-Sectional Study
Source: J Med Internet Res. 2025 Nov 25;27:e72274. doi: 10.2196/72274 (PMC12646552; doi:10.2196/72274)
Supplement: Multimedia Appendix 1 [file jmir-v27-e72274-s001.pdf]

Appendix 1. Information and Communication Technologies (ICTs) and Patterns of eHealth Use Among Older Adults in the Study Sample

Utilization and Frequencies of ICTs	Total (N=2000)
	N (%)
**Technologies Owned [a]**	
Computer (desktop or laptop)	1703 (85.2)
iPad or tablet	1153 (57.7)
Smartphone	1077 (53.9)
Wearable /mobile devices	238 (11.9)
None of the above	168 (8.4)
**Regular Applications Use [a]**	
Phone Text Messaging	917 (50.1)
Video chat	445 (24.3)
E-mail	1654 (90.3)
Facebook	983 (53.6)
Twitter	118 (6.4)
Video games	233 (12.7)
**Internet Use (past 6 months)**	
Yes	1761 (88.1)
No	238 (11.9)
**Frequency of Internet Use (past 6 months)**	
Everyday	1472 (83.6)
A few times a week	180 (10.2)
Once a week	52 (2.9)
2 to 3 times a month	33 (1.9)
Once a month or less often	25 (1.4)

[a] Respondents were asked to specify all technologies owned and applications used (percentages exceed 100%)

Frequency of Internet Use to… [1-5] scale (1=Never; 5=Always)	Median [IQR]
Search for online information about a health problem or condition	3 [2-3]
Self-diagnose yourself when you have a health problem	2 [1-3]
Ask a HC professional questions about your health	1 [1-2]
Access your laboratory test results	1 [1-2]
Access a patient portal/EMR	1 [1-1]
Book a medical appointment online	1 [1-1]
Participate in discussion forums to discuss aspects related to your health	1 [1-1]

Use of mobile applications for health (e.g., smartphone, tablet, iPad)	1<br>[1-1]
**Willingness/Interest in…<br>[1-5] scale (1=Not at All; 5=Totally)**	**Median<br>[IQR]**
Use email to discuss health condition instead of face-to-face	2<br>[1-3]
Obtaining information on trusted websites to use for about health condition	3<br>[1-4]
Having online access to medical records	3<br>[1-5]
