# Supplementary material for: eHealth and the Digital Divide Among Older Canadians: Insights from a National Cross-Sectional Study
Source: J Med Internet Res. 2025 Nov 25;27:e72274. doi: 10.2196/72274 (PMC12646552; doi:10.2196/72274)
Supplement: Multimedia Appendix 2 [file jmir-v27-e72274-s002.pdf]

Appendix 2. Multivariate Analysis Results of Factors Associated with eHealth Use Among Older Adults in the Study Sample

Dependent (Linear Regression)	Independent	Beta	Sig.	95% Confidence Interval (CI) for B	
				Lower Bound	Upper Bound
Search online for information	**Sex (ref: men)**: Women	.148	<.001	0.243	0.472
Adjusted R^2 : 0.077	**Education (ref: elementary):**				
	High school	.171	.006	0.128	0.779
	College degree	.181	.004	0.16	0.814
	University, undergraduate	.230	<.001	0.305	0.965
	University, graduate	.178	<.001	0.279	0.976
	**Income (ref: less than $25,000)**:				
	$25,000-$49,999	-.145	.002	-0.629	-0.14
	$50,000-$74,999	-.122	.007	-0.615	-0.098
	Prefer not to answer	-.141	.001	-0.711	-0.183
	**Region (ref: Prairies)**:				
	Ontario	.075	.028	0.02	0.354
	**Community (ref: Rural <2500 persons)**:				
	Suburb (10,000-50,000 persons)	.130	<.001	0.178	0.549
	**Private Insurance (ref: yes):**				
	No	-.054	.029	-0.248	-0.013
	**Home Care Services (ref: yes):**				
	No	.052	.027	0.04	0.682
	**Pay for quicker access (ref: yes):**				
	No	-.079	<.001	-0.319	-0.083
	**Emergency visits (ref: yes):**				
	No	-.086	<.001	-0.455	-0.14
Self-diagnose Adjusted R^2 : 0.06	**Sex (ref: men)**:				
	Women	.079	.001	0.067	0.282
	**Age (Ref: 65-69 yrs)**:				
	70-74 yrs	-.073	.006	-0.318	-0.054
	75-79 yrs	-.092	<.001	-0.412	-0.117
	85+ yrs	-.089	<.001	-0.646	-0.184
	**Marital Status (ref: single)**:				
	Others/no answer	.062	.010	0.235	1.736
	**Language (ref: French):**				
	English	.098	<.001	0.13	0.378
	**Community (ref: Rural <2500 persons)**:				
	Suburb (10,000-50,000 persons)	.114	<.001	0.123	0.463

	**Pay for quicker access (ref: yes):**<br>No	 <br>-.108	 <br><.001	 <br>-0.362	 <br>-0.142
Ask HC professional Adjusted R^2 : 0.036	**Age (ref: 65-69):**<br>75-79<br>80-84	 .067<br>.104	 .011<br><.001	 0.047<br>0.178	 0.356<br>0.512
	**Education (ref: elementary):**<br>Other	 .121	 <.001	 0.713	 1.866
	**Region (ref: Prairies):**<br>British Columbia	 .062	 .044	 0.006	 0.393
	**Pay for quicker access (ref: yes):**<br>No	 <br>-.073	 <br>.002	 <br>-0.293	 <br>-0.066
Access lab results Adjusted R^2 : 0.127	**Employment (ref: Employed full-time):**<br>Retired	 <br>.100	 <br>.005	 <br>0.126	 <br>0.704
	**Region (ref: Prairies):**<br>British Columbia<br>Ontario	 .290<br>.297	 <.001<br><.001	 0.834<br>0.626	 1.251<br>0.977
	**Community (ref: Rural <2500 persons)**:<br>Suburb<br>Metropolitan city	 <br>.092<br>.077	 <br>.006<br>.030	 <br>0.081<br>0.019	 <br>0.471<br>0.38
	**Private Insurance (ref: yes):**<br>No	 -.055	 .022	 -0.267	 -0.021
	**Pay for quicker access (ref: yes):**<br>No	 <br>-.063	 <br>.006	 <br>-0.297	 <br>-0.049
	**Hospitalizations (ref: yes):**<br>No	 -.063	 .006	 -0.53	 -0.09
Access patient portal/ EMR Adjusted R^2 : 0.061	**Region (ref: Prairies):**<br>British Columbia<br>Ontario<br>Quebec	 .206<br>.132<br>.082	 <.001<br><.001<br>.013	 0.347<br>0.115<br>0.034	 0.628<br>0.352<br>0.291
	**Community (ref: Rural <2500 persons):**<br>Suburb<br>Metropolitan city	 <br>.088<br>.077	 <br>.010<br>.035	 <br>0.041<br>0.009	 <br>0.306<br>0.254
	**Private Insurance (ref: yes):**<br>No	 -.063	 .009	 -0.19	 -0.028
	**Pay for quicker access (ref: yes):**<br>No	 <br>-.105	 <br><.001	 <br>-0.272	 <br>-0.106
	**Hospitalizations (ref: yes):**<br>No	 -.079	 .001	 -0.416	 -0.1

	**Emergency visits (ref: yes):**<br>No	-.058	.020	-0.262	-0.022
Book Appointment Adjusted R^2 : 0.032	**Education (ref: elementary)**:<br>High school<br>College degree<br>University, undergraduate<br>University, graduate<br>Other	 .179<br>.152<br>.153<br>.157<br>.075	 .005<br>.016<br>.015<br>.002<br>.007	 0.119<br>0.065<br>0.071<br>0.169<br>0.192	 0.69<br>0.638<br>0.651<br>0.78<br>1.225
	**Region (ref: Prairies):**<br>Newfoundland and Labrador	-.070	.005	-0.975	-0.176
	**Community (ref: Rural <2500 persons)**:<br>Metropolitan City	.105	.005	0.066	0.368
	**Family Physicians (ref: yes):**<br>No	-.051	.033	-0.503	-0.021
	**Pay for quicker access (ref: yes):**<br>No	-.066	.007	-0.246	-0.039
Participate in discussion forums Adjusted R^2 : 0.007	**Language (ref: French)**:<br>English	.051	.040	0.003	0.141
	**Pay for quicker access (ref: yes):**<br>No	-.055	.020	-0.132	-0.011
	**Perception of health (ref: excellent):**<br>Very Good<br>Good	 -.096<br>-.104	 .012<br>.007	 -0.219<br>-0.228	 -0.027<br>-0.036
**Willingness/Interest in.. [1-5] scale (1=Not at all;5=Totally)**					
Use email to discuss health Adjusted R^2 : 0.126	**Age (ref: 65-69):**<br>75-79 yrs<br>85 + yrs	 -.067<br>-.117	 .006<br>.000	 -0.408<br>-0.808	 -0.07<br>-0.33
	**Education (ref: elementary):**<br>High school<br>College degree<br>University, undergraduate<br>University, graduate	 .122<br>.174<br>.224<br>.196	 .011<br><.001<br><.001<br><.001	 0.083<br>0.256<br>0.43<br>0.504	 0.642<br>0.829<br>1.014<br>1.138
	**Income (ref: less than $25,000):**<br>$25,000-$49,999<br>$50,000-$74,999<br>$75,000-$99,999<br>$100,000-$124,999<br>$150,000 or more	 .075<br>.124<br>.133<br>.066<br>.097	 .043<br>.001<br><.001<br>.022<br><.001	 0.007<br>0.187<br>0.268<br>0.055<br>0.372	 0.448<br>0.673<br>0.805<br>0.7<br>1.177
	**Region (ref: Prairies)**:<br>British Columbia<br>Maritimes	 .087<br>.093	 .002<br><.001	 0.127<br>0.256	 0.551<br>0.801

	**Language (ref: French):**<br>Other	 -.066	 .005	 -1.042	 -0.19
	**Pay for quicker access (ref: yes):**<br>No	 -.135	 <.001	 -0.521	 -0.27
Obtaining information on trusted websites Adjusted R^2 : 0.144	**Age (ref: 65-69):**<br>75-79 yrs<br>80-84 yrs<br>85+ yrs	 -.088<br>-.072<br>-.143	 <.001<br>.004<br><.001	 -0.495<br>-0.46<br>-0.954	 -0.153<br>-0.087<br>-0.471
	**Education (ref: elementary):**<br>High school<br>College degree<br>University, undergraduate<br>University, graduate	 .140<br>.211<br>.233<br>.181	 .003<br><.001<br><.001<br><.001	 0.142<br>0.385<br>0.474<br>0.456	 0.709<br>0.965<br>1.067<br>1.1
	**Marital Status (ref: single):**<br>Others/no answer	 -.048	 .031	 -1.974	 -0.096
	**Income (ref: less than $25,000):**<br>$50,000-$74,999<br>$75,000-$99,999<br>$150,000 or more	 .073<br>.102<br>.082	 .039<br>.003<br>.001	 0.013<br>0.146<br>0.265	 0.508<br>0.693<br>1.082
	**Region (ref: Prairies)**:<br>Maritimes	 .062	 .012	 0.077	 0.638
	**Community (ref: Rural <2500 persons)**:<br>Suburb<br>Metropolitan city	 .079<br>.069	 .008<br>.032	 0.068<br>0.017	 0.454<br>0.373
	**Pay for quicker access (ref: yes):**<br>No	 -.113	 <.001	 -0.467	 -0.212
Accessing online medical records Adjusted R^2 : 0.15	**Age (ref: 65-69):**<br>80-84 yrs<br>85+ yrs	 -.110<br>-.093	 <.001<br><.001	 -0.703<br>-0.819	 -0.272<br>-0.258
	**Education (ref: elementary):**<br>High school<br>College degree<br>University, undergraduate<br>University, graduate	 .107<br>.195<br>.202<br>.141	 .025<br><.001<br><.001<br><.001	 0.046<br>0.389<br>0.432<br>0.33	 0.704<br>1.062<br>1.119<br>1.077
	**Marital Status (ref: single):**<br>Widowed	 -.087	 .023	 -0.666	 -0.049
	**Income (ref: less than $25,000):**<br>$50,000-$74,999<br>$75,000-$99,999<br>$100,000-$124,999<br>$150,000 or more	 .092<br>.114<br>.058<br>.096	 .010<br>.001<br>.039<br><.001	 0.092<br>0.227<br>0.02<br>0.438	 0.664<br>0.861<br>0.78<br>1.385

	**Region (ref: Prairies):**<br>British Columbia<br>Maritimes<br>Quebec	 .083<br>.089<br>.120	 .002<br><.001<br>.005	 0.14<br>0.275<br>0.136	 0.639<br>0.927<br>0.766
	**Community (ref: Rural <2500 persons)**:<br>Small town<br>Suburb<br>Metropolitan city	 .064<br>.128<br>.123	 .019<br><.001<br><.001	 0.048<br>0.266<br>0.198	 0.537<br>0.714<br>0.611
	**Live in (ref: my home):**<br>A retirement home<br>Other	 -.049<br>.051	 .025<br>.015	 -0.793<br>0.466	 -0.053<br>4.388
	**Pay for quicker access (ref: yes):**<br>No	 -.084	 <.001	 -0.441	 -0.147
**Use of.. [1-5] scale (1=Never;5=Always)**					
mApps for health Adjusted R^2 : 0.029	**Age (ref: 65-69):** 85+ yrs	-.061	.018	-0.367	-0.035
	**Region (ref: Prairies):** British Columbia	.064	.036	0.01	0.281
	**Community (ref: Rural <2500 persons)**:<br>Metropolitan City	 .135	 <.001	 0.103	 0.336
	**Perception of health (ref: excellent):**<br>Good<br>Fair	 -.074<br>-.073	 .049<br>.023	 -0.251<br>-0.322	 0<br>-0.023
**Dependent (Logistic Regression)**	**Independent**	**Exp (Beta)**	**Sig.**	**95% Confidence Interval (CI) for B**	
				**Lower Bound**	**Upper Bound**
FDT (yes/no)	**Live in (ref: my home):**<br>A retirement home	 0.366	 0.033	 0.145	 0.923
	**Home Care Services (ref: yes):**<br>No	 3.427	 0.002	 1.550	 7.596
	**Emergency visit (ref: yes)**:<br>No	 2.16	 0.008	 1.228	 3.800
